# Assessment of disease activity in Graves’ orbitopathy

**DOI:** 10.3389/fopht.2025.1697892

**Published:** 2025-11-26

**Authors:** Arnaud R. G. G. Potvin, Ioana C. Lacraru, Peter H. Bisschop, Maartje M. L. de Win, Anja K. Eckstein, Peerooz Saeed

**Affiliations:** 1 Orbital Center, Department of Ophthalmology, University Medical Center, University of Amsterdam, Amsterdam, Netherlands; 2 Department of Endocrinology, University Medical Center, University of Amsterdam, Amsterdam, Netherlands; 3 Department of Radiology and Nuclear Medicine, University Medical Center, University of Amsterdam, Amsterdam, Netherlands; 4 Department of Ophthalmology, Medical Faculty, University Duisburg-Essen, Essen, Germany

**Keywords:** Graves’ ophthalmopathy, MRI, disease activity, diffusion weighted imaging, short tau inversion recovery (STIR), TSH receptor antibodies (TRAb), biomarker

## Abstract

**Purpose:**

Graves’ orbitopathy (GO) is the most common extra-thyroidal manifestation of Graves’ disease (GD). Clinical disease activity and severity stage at the time of diagnosis are commonly used to determine the optimal treatment. There are still controversies regarding the “gold standard” for establishing disease activity. Although the clinical activity score (CAS) is the best evaluated parameter and therefore widely used, it lacks the ability to predict disease progression in all patients and response to anti-inflammatory treatment. Additional predictors are needed to select the optimal treatment for each patient.

**Methods:**

We conducted a comprehensive review of the literature on activity assessment in GO.

**Results:**

A large variety of parameters are used in studies to assess disease activity, most common clinical activity/severity scores, orbital imaging techniques, and serum biomarkers. CAS remains the best validated way for activity scoring. Other promising parameters, including specific MRI sequences (short tau inversion recovery, T2 mapping, and diffusion-weighted imaging sequences) and serological biomarkers [thyroid-stimulating immunoglobulin (TSI)/thyroid-stimulating hormone (TSH)-binding inhibitor immunoglobulin (TBII)], are starting to prove their utility in quantifying disease activity and in predicting the outcome of GO. TBII and TSI measurements’ cutoff values for prognostic statements are available for almost all routine test systems and should be used more systematically as biomarkers for GO.

**Conclusions:**

CAS is still considered the gold standard for assessing disease activity. Applied alone, CAS fails to predict disease progression in all patients. The future assessment is probably a combination of clinical, serological, and imaging measurements. The selection of treatment should be tailored to the manifestations and main treatment effects of the available drugs. Future studies will need to determine which parameters provide the best predictions for each drug class.

## 1 Introduction

Graves’ disease (GD) is an autoimmune disorder primarily involving the thyroid gland and orbit. Its pathogenesis is associated with the abnormal production of anti-thyroid-stimulating hormone receptor (TSHR) antibodies (TRAbs) that bind to the TSHR. Most of these antibodies stimulate the thyroid, inducing hyperthyroidism (1). Graves’ orbitopathy (GO) represents the most frequent extra-thyroidal manifestation. How this entity develops has not been fully elucidated, but it involves inflammatory and proliferative pathways. Orbital fibrocytes with TSHR and CD40 expression play a central role (2). Stimulation by TRAbs of the receptor complex formed by TSHR and insulin-like growth factor 1 receptor (IGF1R) induces inflammatory cytokine release, attracting more immune cells (3), as well as glycosaminoglycan (GAG) production and adipogenesis, leading to the remodeling of retro-orbital soft tissues, such as the expansion of extraocular muscles and adipose tissues (4, 5). Approximately 15%–40% of patients with hyperthyroidism develop mild GO, and approximately 5% progress to moderate-severe GO, of which 5%–9% will develop dysthyroid optic neuropathy (DON) (6–9). Although there is a decline in the prevalence of late-stage GO, the natural evolution of self-limitation over time of GD and GO is still unclear (10).

Before the dawn of IGF1R blocking agents, mainly immunomodulators were used to treat GO, and they still are the mainstay in many countries (11). The indication for immunomodulatory treatment was based on inflammatory activity, as this is the manifestation that responded best to immunomodulation. With the approval of teprotumumab, a human monoclonal antibody directed against IGF1R, by the Food and Drug Administration (FDA) and now the European Medicines Agency (EMA), the indication for treatment is not only based on clinical activity anymore. Clinically inactive patients with progressive proptosis and motility impairment respond significantly to the new therapy as well. Even years after the first manifestations, a therapeutic response can be observed. Additional biomarkers beyond signs of inflammation, which show more active proliferation, are needed. The new American Thyroid Association (ATA)/European Thyroid Association (ETA) guidelines include an adjusted list of indications to treat according to dominant manifestations (12).

The term “disease activity” in GO has historically been used interchangeably to describe the inflammatory phase, likelihood of progression, or likelihood of response to immunosuppression. However, these concepts, while related, are not identical. There is a shift ongoing in the concept of disease activity, where currently many experts believe it should be regarded as the presence of ongoing, potentially reversible orbital inflammation capable of driving clinical change and responding to therapy. Consequently, accurate dynamic clinical findings, serological and imaging biomarkers, and functional changes should be thoroughly investigated in order to improve our understanding of disease activity and, hence, the therapeutic outcome (13). This review aimed to provide an overview of parameters to quantify disease activity and to predict the disease progression in GO and highlighted the importance of considering not only the clinical activity score (CAS) but also imaging parameters, TRAb levels, and control of thyroid function for activity and progression assessment.

## 2 Methods

We conducted a review of the literature from the electronic PubMed database, which included randomized controlled, observational clinical trials, book chapters, and reviews with a focus on the assessment of activity and prognostic factors in GO until May 2025. We documented the most relevant studies on novelties in orbital imaging and serological bioassays. Our strategy was based on the following search terms: Graves’ orbitopathy/ophthalmopathy, thyroid eye disease/thyroid associated ophthalmopathy, imaging, magnetic resonance imaging (MRI), scintigraphy, disease activity, clinical activity score, predictive/prognostic factors, TSH receptor autoantibodies, and therapy response. We also went through the reference lists of articles and selected relevant articles. From those results, 167 papers describing quantitative and qualitative measurements assisting in the diagnosis, activity assessment, and staging of GO based on imaging (MRI and scintigraphy) and serological markers (TRAb analysis) were selected.

## 3 Results

### 3.1 Clinical scores and classifications of disease activity

The ATA structured clinical features under the mnemonic NOSPECS, but it has no utility in the assessment of disease activity (14). The concept of activity in GO started from the hypothesis of two different disease stages according to Rundle’s curve, characterized by a biphasic course, specifically the dynamic, inflammatory phase followed by the static, quiescent phase (15). The distinction between the active and inactive states can be unclear, and patients often remain with ocular sequelae (16). The CAS, a simple 7-point classification (10 points post-presentation) based on clinical inflammatory signs, was proposed in 1989 and used to categorize patients as active or inactive (17). The validated 10-point score predicted the therapeutic outcome after oral prednisolone or radiotherapy with a positive predictive value of 80% (18). At the first visit, patients are categorized as active if CAS equals or is higher than 3 (17). In the 2021 European Group on Graves’ Orbitopathy (EUGOGO) guidelines, a revised five-item composite index, which includes pain with eye movements, painful retrobulbar pressure, eyelid swelling, conjunctival hyperemia, and chemosis, is used to assess treatment response (11, 19). There are some problems with CAS, however. For example, the authors reported that 36% of patients with a CAS < 4 were still considered responders (18). In addition, CAS scoring is influenced by race, considering that it tends to be lower in Asians despite signs of active disease (20). In addition, it does not reflect subclinical or compartmental inflammation. Therefore, the question remains if there are other parameters complementary to CAS that can improve activity scoring (18).

In addition to the NOSPECS classification, CAS, and EUGOGO severity scale, another protocol was developed in 2006 by Dolman and Rootman to document the global severity grade alongside disease activity. The VISA system grades activity and severity of each clinical parameter independently, namely, vision (DON), inflammation, strabismus, and appearance (21). The “I” score is considered the equivalent of CAS: caruncular edema (0–1), chemosis (0–2), conjunctival redness (0–1), lid redness (0–1), lid edema (0–2), retrobulbar ache (0–2), and diurnal variation (0–1). Since it allows quantification in three parameters and includes diurnal variation, it may reflect the activity even better than a purely binary score like CAS, but both scoring systems have not been compared directly (21). A VISA grading atlas improves interrater reliability in the photographic assessment of GO (22). However, VISA has not been prospectively validated as a prognostic tool, while CAS has (11, 18). Although VISA allows for comprehensive, reliable clinical recording and communication, it should be used alongside CAS rather than as a replacement for it.

### 3.2 Clinical modifiable factors associated with treatment response to anti-inflammatory treatment

These classification systems offer a way to estimate which patients will respond well to anti-inflammatory treatment, but there is still a group of non-responders, on the one hand, and inactive patients that actually improve with treatment, on the other hand. To refine the selection of patients for therapy, other factors need to be considered.

#### 3.2.1 Smoking

The detrimental influence of smoking on GO has been well established. It increases the risk of GO occurrence as well as the severity (23, 24). Smokers have a higher risk of developing moderate-to-severe GO, and some studies have also associated it with an increased risk of DON. Smokers require decompression and steroids more frequently than non-smokers, even though they are, on average, significantly younger than non-smoking patients with DON (23, 25, 26). It was recently suggested that combustion rather than nicotine drives the risk and complications of GO, an important finding as alternatives like electronic cigarettes are on the rise (27). They also have a lower and slower response to intravenous glucocorticoids (IVGCs) as well as orbital radiotherapy (19, 28–30). Even past smoking was an independent risk factor for poor response to IVGCs, and there was a similar trend for passive smoking in a multivariable prediction model correcting for age, sex, body mass index, CAS, TRAbs, GO duration, and proptosis (29). Non-smoking was not found to be independently associated with CAS inactivation after IVGC treatment followed by oral mycophenolate (31). More recently, a single-center retrospective cohort reported attenuated improvements in CAS, diplopia, and proptosis in smokers treated with teprotumumab versus non-smokers, including a significantly smaller reduction in proptosis (32). These findings show that smoking is a consistent negative predictor of treatment response in active GO, although for some second-line biologics, like tocilizumab or rituximab, there have been no smoking-stratified or smoking-adjusted treatment-response analyses.

#### 3.2.2 Thyroid status and disease duration

Achieving a euthyroid state promptly is considered paramount in the treatment of GO, as both hyper- and hypothyroidism negatively impact GO (11, 33–35). For IVGCs, multiple cohorts have suggested that shorter disease duration is favorable and that high TSI/TRAbs rather than FT4/TSH tracks poorer response (36–38). Treatment within ≤6–12 months from onset and a euthyroid status have also been found to be favorable for orbital radiotherapy. For teprotumumab, pooled randomized controlled trial (RCT) and extension data indicate robust efficacy irrespective of baseline disease duration (including longer-duration cohorts), with no consistent signal that FT4/TSH modifies response (39, 40). For mycophenolate, which is usually combined with IVGCs and other biologics, like rituximab and tocilizumab, the literature rarely reports FT4/TSH-stratified outcomes. However, shorter disease duration plausibly contributes to better outcomes (41, 42).

#### 3.2.3 Combined approach

All grading systems play a critical role, even though they cannot predict clinical activity, evolution, and therapeutic outcome with certainty. Therefore, a combined protocol based on the previously described classifications and serological tests could guide the physicians in a more straightforward GO assessment and management (43, 44). There has been considerable effort in elucidating risk factors for the manifestation and progression of GO. Smoking, high TRAb levels, and poor control of thyroid function have been repeatedly pointed out. Other factors that may have the same strong influence are more challenging to measure, like the status of the immune system and psychological factors. The results suggest that a composite score is likely to be the best predictive tool. However, a CAS higher than 1 was associated with the highest odds ratio (4.45) for the development of an overt GO. Therefore, one can assume that sensitive imaging techniques may be useful in the prediction of the GO course and could guide the physicians in a more straightforward GO assessment and management.

### 3.3 Magnetic resonance imaging

Among the wide spectrum of imaging modalities in the assessment of GO, MRI may play an essential role in quantifying the inflammatory activity due to the ability to evaluate the presence or absence of water, and thus edema and inflammation, in the orbital soft tissues (45). In the orbit, it is important to use a fat-suppression technique, like Dixon, iterative decomposition of water and fat with echo asymmetric and least squares estimation (IDEAL), or fat fraction mapping, to distinguish the hyperintense edema/inflammation from the hyperintense orbital fat. In general, T1- and T2-weighted MRI sequences are the most frequently used techniques for evaluating active disease, although short tau inversion recovery (STIR) has gained attention in more recent studies. In all three sequences, active disease is characterized by inflammatory edema, which leads to increased signal intensity (SI) in affected orbital tissues (46). This edema is also associated with prolonged T1 and T2 relaxation times (T1/T2RT) in the orbit (47). In addition to improved diagnosis, imaging is increasingly playing a role as a guide for management, as several imaging biomarkers have been investigated as predictors of therapeutic outcome. The advent of biologicals in TED therapy emphasizes the importance of personalized treatment approaches, as various agents act on different disease features and may yield differential benefits across patient profiles (12). Therefore, integrating clinical assessments with MRI-derived data could enhance diagnostic accuracy in cases of GO.

#### 3.3.1 Short-tau inversion recovery

STIR is a fat-suppression technique that is relatively independent of field inhomogeneities and can be complemented by T1 and T2 contrast (48). It allows the sensitive detection of inflammatory edema in the orbit. Beyond correlating with CAS, several studies have demonstrated the predictive potential of STIR-derived SI ratios (SIRs) for treatment response to IVGCs in GO.

**Figure 1:**
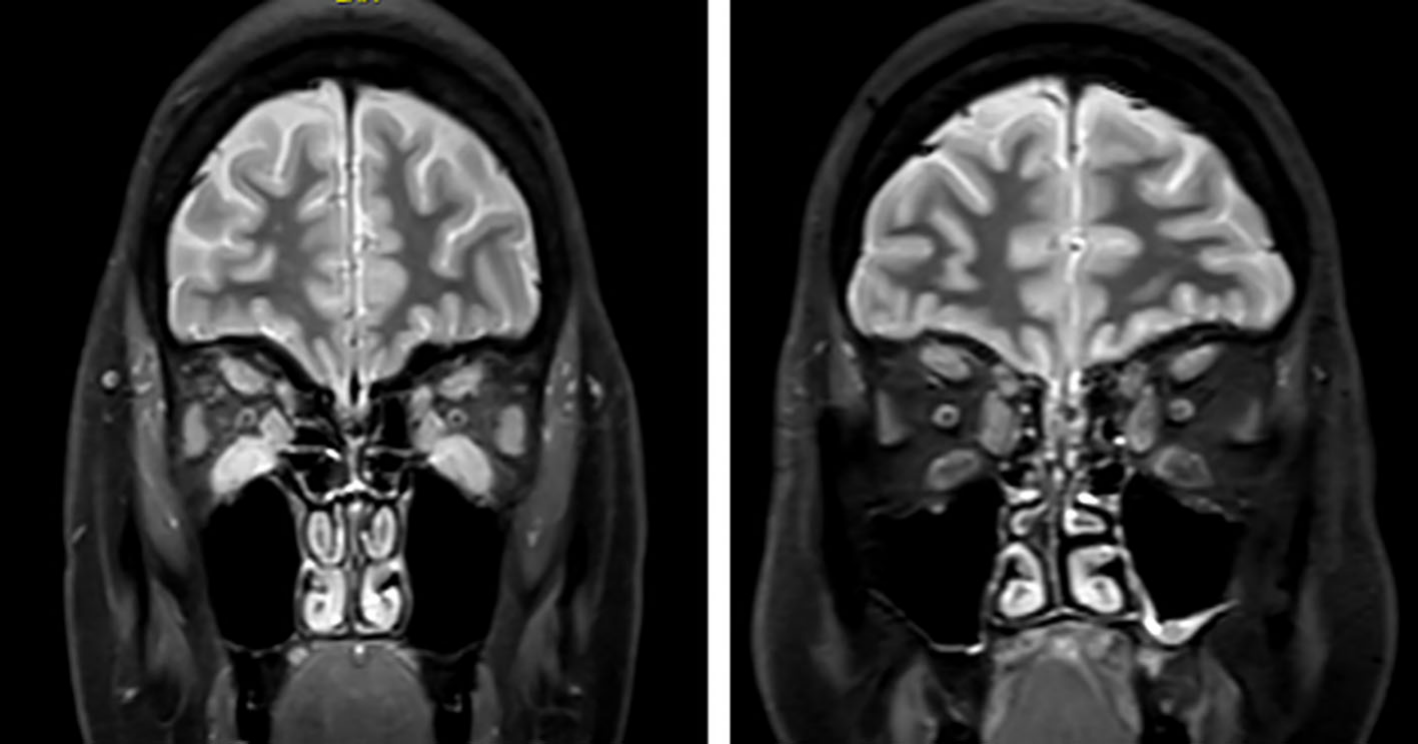
Left: coronal STIR MRI in a patient with active disease. Increased and infiltrated orbital fat tissue; thickening of all EOMs with edematous changes; no apical crowding. Right: same patient after intravenous glucocorticoid therapy. Proptosis decreased from severe to moderate on both sides clinically (not shown); normal fat tissue; fatty infiltration of EOMs with no edematous changes. STIR, short tau inversion recovery; EOMs, extraocular muscles.

**Figure 1** shows severe enlargement of extraocular muscles (EOMs) and edematous changes in a patient with active GO and a CAS of 6 on the left and the follow-up MRI after IVGC treatment on the right, which reveals the decrease in thickness and the fatty infiltration of all EOMs. Pre-therapy medial and inferior rectus SIRs on both contrast-enhanced T1 and on STIR decreased significantly after IVGC treatment (49). Similar results were found in other studies, showing a decrease in SIR in EOMs after IVGC treatment, and higher baseline SIRs have been associated with a better response (49, 50). Post-therapy EOM SIR higher than 2.5 is associated with worsening of GO, despite a low CAS (50). In a recent study, a score based on STIR and EOM enlargement before and after 6 g of IVGCs was used to guide IVGC cumulative dose. If the score after treatment was not reduced, an additional 3 g of IVGC was administered. The MRI score after 6 g of IVGC was the only parameter associated with disease recurrence within 1 year (51). These results suggest that STIR-derived parameters could be useful for predicting therapeutic response and recurrence risk and for individualizing treatment dosing.

Earlier studies have established that the mean STIR SIR of the EOMs compared with the temporalis muscle correlates positively with CAS and NOSPECS severity grading, and negatively with ocular motility, suggesting that motility restriction may reflect active inflammation (46). When comparing EOMs on STIR and T1 sequences, the difference in cross-sectional areas may indicate perimuscular edema (19, 27). STIR and contrast-enhanced T1-weighted sequences are both effective in detecting edema of the EOMs (28). Quantitatively, the medial rectus SIR (compared with the temporalis muscle) can discriminate active from inactive disease with an area under the curve (AUC) of 0.905 at a cutoff of 2.23 (29), while the inferior rectus SIR (compared with white matter) shows an optimal cutoff value of 2.9 (30). Alongside EOM assessment, the SIR of orbital fat on STIR and T2 sequences is significantly higher in clinically active patients compared with inactive patients and controls, and correlates with EOM SIRs and CAS (31).

In summary, STIR provides a sensitive, operator-independent assessment of orbital inflammation. While correlations with CAS support its biological validity, one must be careful not to let this be the only factor in evaluating a marker’s validity for clinical use as a tool to assess activity. The longitudinal evidence indicates that STIR-derived SIRs may serve as predictive imaging biomarkers for treatment response and disease recurrence in GO, supporting their potential as a future assessment tool for disease activity.

#### 3.3.2 T2 mapping, IDEAL, and Dixon

T2RT represents the decay rate of the MRI signal and depends on the water content within a tissue. Increased water content within EOMs reflects inflammation, resulting in prolonged T2RT. Conventional fat-suppressed T2-weighted imaging techniques, such as inversion recovery, are susceptible to artefacts caused by magnetic field inhomogeneity, particularly at the tissue–air interface between the sinuses and the orbit, although recent techniques mitigate these limitations (52). In contrast, Dixon MRI employs chemical shift-based fat suppression, allowing direct separation of fat and water signals. Studies have suggested that Dixon outperforms conventional methods in terms of image quality and uniformity of fat suppression (53–55). When combined with fat fraction (FF) mapping, T2 relaxation mapping allows differentiation between the water component representing active inflammation and the fat component reflecting chronic tissue remodeling (56).

As for STIR, T2RT can be used to predict and monitor treatment response to IVGCs (57, 58). Maximal, mean, and the top 10% T2RT values of EOMs, as well as the EOM water fraction, are independent predictors of responsiveness (58, 59). Both affected and apparently unaffected GO EOMs showed a significant post-treatment reduction in T2 SIR and T2 histogram parameters such as entropy and inhomogeneity, suggesting both clinical and subclinical response and showing that these specialized techniques can detect early or subclinical EOM involvement (60). Comparing conventional T2 mapping of the EOMs with fat-suppression T2 mapping, although both techniques can distinguish active from inactive disease (61), the latter has been found to be superior (62), also in terms of predicting IVGC response (63). These findings support T2-based techniques as reliable imaging biomarkers for therapeutic monitoring and prediction of treatment outcome.

T2 mapping also enables a more sensitive assessment of disease activity compared to clinical scores. Maximum (T2RT_max_) and mean (T2RT_mean_) EOM T2 values differ significantly between active and inactive disease (57, 64) and also between patients with CAS 0 and CAS 1–2, suggesting that disease staging determined only by CAS could underestimate inflammatory activity. These “CAS-negative” patients with prolonged T2RT have been observed to improve after immunosuppressive therapy (65). T2RT measured from a single-slice region of interest (ROI) is more accurate than from a solitary hotspot ROI (64). In an initial feasibility study, T2RT was significantly higher in GO patients compared to healthy controls, while FF was also increased but to a lesser extent. Both T2RT and STIR SIR decreased after IVGC treatment, confirming their responsiveness to treatment. Notably, in some cases, T2RT remained prolonged despite clinical improvement, suggesting that T2 mapping may reveal persistent subclinical inflammation beyond CAS evaluation (56).

Additional morphological information may further refine disease staging. A prolonged T2RT together with an increased EOM thickness may point toward edematous changes (65), while a diffuse or patchy low-intensity signal may indicate fibrosis. In patients with restrictive diplopia, a diffuse low-signal pattern on T2 has been associated with poor reversibility after medical treatment (66).

The lacrimal gland (LG) could also provide clues toward active disease. ROI T2 mapping and apparent diffusion coefficient (ADC) values could determine disease activity alone and in combination with clinical parameters (67). The coronal LG area and T2 SIRs are increased in active compared to inactive GO patients, and their enlargement positively correlates with CAS, with a T2 SIR of 2.57 representing the threshold for activity reported in one study (68).

Taken together, T2 mapping, especially with fat-suppression techniques like Dixon or IDEAL, can be a useful, objective tool to assess inflammation in the orbit and offer a predictive value for IVGC response, early treatment monitoring, and identification of subclinical disease activity, extending its utility beyond correlation with CAS.

#### 3.3.3 T1-weighted imaging

T1 mapping is able to estimate the severity of fibrosis in tissues and is valuable for the evaluation of the response in GO patients with diplopia after IVGC treatment. Lower pre-treatment T1 mapping signals could indicate the prognosis of refractory diplopia for both active and inactive patients (69). A CAS > 4 positively correlates with SIRs on T2 turbo inversion recovery magnitude sequences as well as on contrast-enhanced T1 sequences. The difference in SIRs between the T1-SIR and T2-SIR could be an indicator of the type of edema, in the sense that a large SIR difference would signify an inflammatory edema, while a small one could signify only congestion (70). Higher signals are observed on mean T2 and contrast-enhanced T1 in the EOMs with clinical impairment compared to EOMs without. In addition, higher T2 SIRs are reported in EOMs without symptomatic dysfunction in GO patients compared with healthy control EOMs (71). Recent literature has coined LG herniation as a new potential MRI biomarker for the assessment of disease activity, as the proptosis and the degree of LG herniation measured on contrast-enhanced axial T1 with fast spin-echo fat saturation are higher in active compared with inactive GO (72). In addition, the ratio of LG herniation to orbital fat thickness was an independent predictor of response to treatment with glucocorticoids in active, moderate-to-severe GO (73).

GAG chemical exchange-dependent saturation transfer (GagCEST) is a new T1-based technique that detects the relative content of GAG molecules and does not require contrast. It has shown promising results in the evaluation of knee cartilage (74) but requires advanced 7T MRI scanners to function optimally (75). It could be interesting to visualize GAG content in more detail, as it is related to IGF1R-driven volume increase and may help to guide treatment choice in the future, but GagCEST has not been studied in the orbit yet.

#### 3.3.4 Diffusion-weighted imaging and diffusion tensor imaging

**Figure 2:**
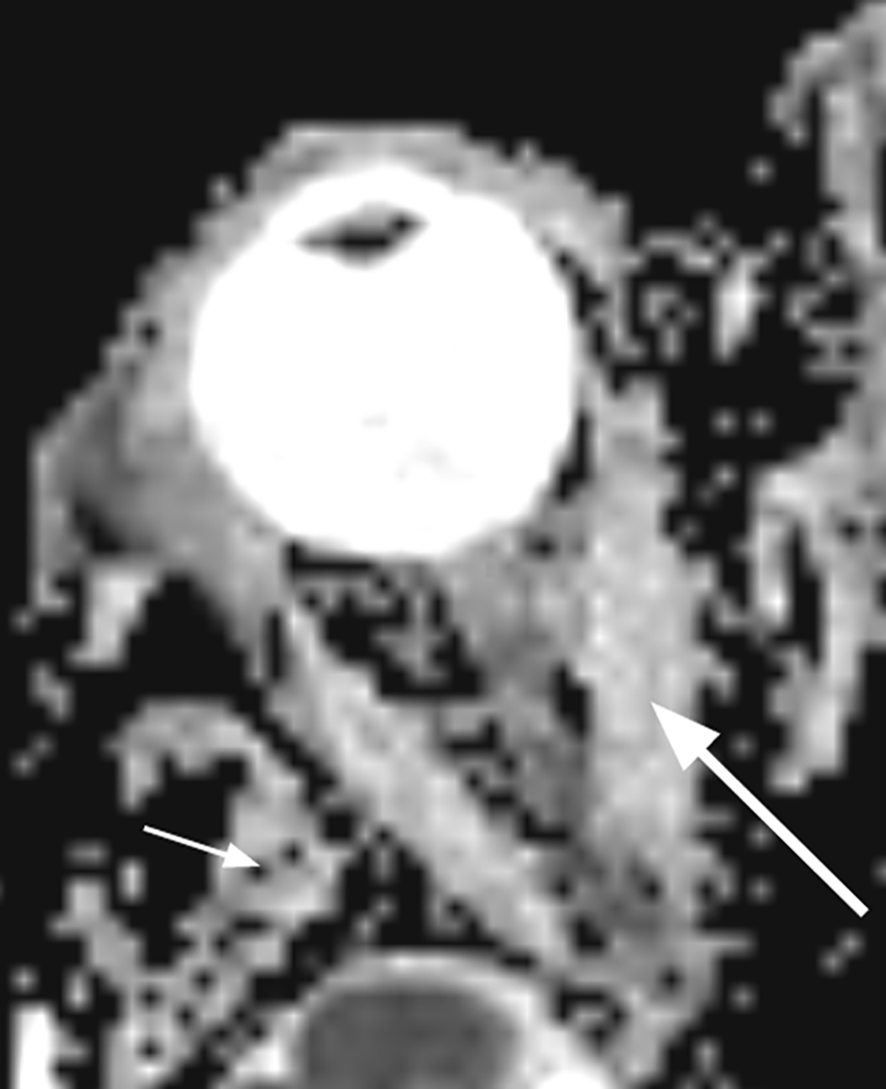
DWI image. To standardize measurements across scanners, a ratio between the ADC of a ROI in the temporalis muscle and the ADC of ROIs in the rectus muscles can be calculated. Small arrow, temporalis muscle; large arrow, medial rectus muscle. DWI, diffusion-weighted imaging; ADC, apparent diffusion coefficient; ROI, region of interest.

Diffusion-weighted imaging (DWI) and diffusion tensor imaging (DTI) rely on the movement of water molecules in the body in relation to the tissue density; i.e., the higher the cellularity, the more restricted the motion will be (76). Classic metrics include ADC, fractional anisotropy (FA), and mean diffusivity (MD). **Figure 2** shows a DWI scan on the left and two ROIs, one in the medial rectus and one in the temporalis muscle, on the right, with which a ratio in ADC values can be calculated to allow standardization between scans.

Beyond correlation with clinical scores, DWI has demonstrated predictive value for treatment response and longitudinal disease monitoring. In a prospective study using non-echo-planar DWI, both pre- and post-therapy ADC values of the EOMs were shown to decrease after IVGC treatment, and this reduction correlated with the clinical improvement of ocular motility (77, 78). Patients with persistently high post-treatment ADC exhibited ongoing or recurrent disease activity despite low CAS, suggesting that DWI can reveal subclinical inflammation and predict treatment outcome (77, 78). Moreover, DWI allows quantitative follow-up without requiring precise ROI measurements, making it practical for serial monitoring of therapy response (78).

Predictive and subclinical detection capabilities are further supported by the observation that elevated ADC values are found even in non-enlarged or clinically unaffected EOMs when compared to controls (71). High EOM ADC values in patients with low CAS may precede clinical manifestations, and improvement in ocular motility after immunosuppressive therapy confirms that DWI detects early reversible inflammation (77–79). The results comparing non-echo-planar DWI with STIR SIR values revealed a balanced interplay between the two techniques, demonstrating good agreement in both pre- and post-treatment assessments. Taken together, these findings indicate that DWI can detect occult or asymptomatic EOM involvement not visible on routine MRI, possibly facilitating early diagnosis, the identification of patients at risk of progression, and the prevention of irreversible sequelae (71, 79).

Mean EOM ADC is significantly higher in active compared with inactive and control groups (77, 78, 80, 81). ADC in the superior rectus relative to the temporalis muscle has been found to be an independent discriminator between moderate-to-severe GO and DON patients, possibly expanding the utility of DWI to the prediction of visual deterioration (82). Not only general inflammation in EOMs but also the distribution of inflammation can provide valuable information. Histogram-based parameters, including ADC entropy and standard deviation, are higher in active GO and correlate with CAS, indicating more heterogeneous inflammation in active disease. Higher ADC and more uneven distribution of inflammation, therefore, indicate disease activity (83, 84). Focusing on other orbital structures, LG ADC is positively correlated to CAS and differs significantly between GO patients and controls (85); uni- and multivariable regression models confirm ADC as an independent discriminator of active and inactive disease (67).

DTI is capable of providing quantitative data on microstructural changes of orbital tissues (86). Lower FA and higher MD, axial diffusivity (AD), and radial diffusivity (RD) of EOMs and LGs were found in GO compared to healthy controls, especially in moderate-to-severe GO (86–88). These findings reflect fiber disorganization and increased extracellular water content. Region-specific analyses show that the mean ADC of the inferior rectus muscle, but not the other EOMs, differs significantly between active and inactive disease, while the ADC range (max–min) within each EOM inversely correlates with CAS and also differs between active and inactive disease. Both parameters have comparable diagnostic power (84). Together, these DTI parameters offer quantitative insights into the microstructural alterations of EOMs and LGs in GO, potentially enhancing disease characterization and monitoring.

DTI reveals important functional details on the axonal integrity at the level of the optic nerve. Higher FA is measured in GO patients compared to the control group, and additionally, FA correlates with both CAS and EOM thickness and is significantly higher in active compared with inactive GO (89). These DTI parameters of the optic nerve could be useful to aid the diagnosis of DON, as was proposed by one study, which also found correlations between AD, RD, FA, and MD of the optic nerve with CAS and visual acuity (90). Interestingly, all these indices change before the onset of DON, offering an essential tool for early detection and prognostication (89).

In summary, DWI and DTI provide quantitative, non-invasive biomarkers of orbital inflammation and microstructural integrity. DWI can not only predict and monitor therapeutic response to IVGC but also detect subclinical or residual inflammation beyond clinical activity scores and could be helpful in the diagnosis of DON, while DTI offers insight into disease activity in addition to early indicators of optic nerve involvement and microstructural changes relevant for DON risk stratification.

#### 3.3.5 Considerations regarding teprotumumab treatment

Reduced EOM volume is one of the hallmarks of teprotumumab treatment, leading to decreased proptosis and improved motility and diplopia (91–93), and it has also been observed in a proof-of-concept RCT with batoclimab, an inhibitor of the neonatal fragment crystallizable receptor (FcRn) (94). Total EOM and fat volume reduced significantly, resulting in EOM volumes similar to a normal population (92). A prospective study following these preliminary results demonstrated lower reductions of EOMs (33% vs. 23%) and fat volume (29% vs. 9%) (95). Reductions are observed in all recti, leading to a decreased total EOM to orbit cross-sectional area (93). Proptosis response is predicted by higher baseline proptosis and larger EOM volumes, or lower fat-to-muscle ratio, and is not associated with age, gender, smoking, diplopia at baseline, disease duration, or CAS (95, 96). Lower impact on fat volume also means that patients with a higher fat-to-muscle ratio may benefit from decompression surgery more than teprotumumab infusions, highlighting how important imaging, whether it be CT or MRI, is for management decisions (97).

### 3.4 Scintigraphy

Receptor-based imaging techniques have gained importance in the assessment of clinical activity and in predicting the therapeutic outcome for GO patients. The principle relies on the ability of radioisotopes to seek inflamed or necrotic tissues. More precisely, a specific metabolite or ligand for a receptor is coupled to a radioisotope to allow *in vivo* imaging. The positive uptake in Graves’ patients is due to the upregulation of receptors found at the level of orbital fibroblasts and lymphocytes, which trace the retrobulbar inflammation (16). This approach offers a molecular-level view of disease activity that complements anatomical imaging techniques.

The orbital/occipital uptake of octreotide, a synthetic somatostatin analogue, may predict the patients’ outcome after radiotherapy, as responders were associated with a higher uptake compared with non-responders (98, 99). Regarding ^99m^Tc-hydrazinonicotinyl-Tyr3-octreotide (^99m^Tc-TOC) scintigraphy orbital/occipital ratio, it has a positive predictive score of 88.9% and correlates with CAS, just like the newly developed tracer ^99m^Tc-P829 (100, 101). Diethylenetriaminepentaacetic acid (DTPA) and indium-111-pentetreotide scintigraphy are useful in evaluating the efficacy of corticosteroid therapy, defined by a decrease in CAS, and in selecting suitable candidates for radiotherapy, given the significant difference between the uptake value before and after treatment and the responsiveness to therapy in patients with high initial DTPA uptake with a positive predictive value of 76% (102–104). Image fusion analysis at the level of the LG, an important anatomical landmark with somatostatin receptors, reveals a high uptake of ^99m^Tc-TOC. The increased LG uptake, responsible for the so-called “eye glass” scintigraphic pattern, may be the underlying factor for the clinical sign of eyelid swelling (105). With the use of anti-TNF-α labeled with ^99m^Tc or ^99m^Tc(V)-dimercaptosuccinic acid (DMSA), patients with a clinically inactive stage could present positive scintigraphic signs and raise the question of subclinical disease (106). Nonetheless, ^99m^Tc-anti-TNF-alpha scintigraphy has a very low positive predictive value of 68.4% but a high negative predictive value of 96.7% in evaluating active orbital inflammation (107). Altogether, these radiotracers provide promising yet variable diagnostic value, highlighting the importance of choosing the right tool for specific clinical questions.

A novel observation is the increased level of calsequestrin, an extraocular muscle antibody, in active compared with inactive patients and its positive correlation with CAS, but evidence regarding the prediction of therapeutic response is lacking (108). Comparing the “gold standard” of detecting orbital inflammation, namely, MRI T2RT score, with receptor-based scintigraphy, all imaging techniques present a balanced interplay (109). Although MRI has the highest sensitivity of 0.93, Single Photon Emission Computed Tomography (SPECT)/CT has the highest specificity of 0.89, with an increased uptake even in patients with undetectable activity signs on MRI and low CAS. Alongside MRI techniques, scintigraphy could be, in theory, a supplementary tool in the assessment and diagnostic algorithm of GO and in monitoring GO patients before and after immunotherapy or external radiation therapy (110, 111). However, in our opinion, due to the high costs of the tracer and the fact that scintigraphic technology does not have more significant results than MRI, these studies remain rather theoretical and have no practical value in the routine evaluation of GO.

### 3.5 Serum biomarkers

#### 3.5.1 TRAbs

##### 3.5.1.1 Assay technologies

Many studies have focused on measuring TRAbs in order to discover new diagnostic criteria for active disease and to prove their usefulness as biomarkers of GO (112, 113). According to the ETA, TRAbs are a sensitive measurement for accurately diagnosing GD and its extra-thyroidal manifestations (114).

TRAbs can be measured in different ways, and the most widely applied are competitive binding immunoassays, also named TSH-binding inhibitor immunoglobulin (TBII) assay, using mainly M22 monoclonal antibody (some also labeled TSH), which binds the TSHR with high affinity. This technique measures the concentration of circulating antibodies and does not differentiate between stimulating (TSI), neutral, or blocking antibodies (TBAbs). Other assays aim to measure specifically the stimulating activity of TRAbs (113, 115). An automated chemiluminescent assay employs, using a bridge model, a pair of recombinant human TSHR chimeras aiming to capture and detect TSI by offering only the region of TSHR where preferably stimulating TRAbs bind. A more elaborate bioassay system with genetically engineered Chinese hamster ovary cell lines (CHO Mc4), which contain chimeric human TSHR linked to a luciferase gene, can measure stimulating and blocking antibodies (116).

##### 3.5.1.2 Diagnostic sensitivity and specificity

The presence of TRAbs is presumed to be highly specific for the diagnosis of GD, confirming the diagnosis. The diagnostic sensitivity/specificity is comparable for the newest generation of binding competition assays, bridge assays and bioassays, and is nearly 100% (117–120). During the course of the disease, the sensitivity of the bioassay is higher, as antibodies can be traced for a longer time in comparison with binding competition assays and bridge assays (121, 122).

##### 3.5.1.3 Correlation with activity and severity of GO

Seo et al. (2018) summarized that disease duration, the associated treatment for the underlying hyperthyroidism, and even the patients’ nationalities may influence the TRAb levels and thus their interpretability (123). This explains the heterogeneity of the correlation result. In incidence cohorts, it has been shown that TSI levels correlate closely with activity and severity (124). The same has been shown for the subgroup of patients with GO and Hashimoto thyroiditis (125).

##### 3.5.1.4 Prediction of the course of GO

Stöhr et al. (2021) concluded in a recent study that the predictive power of the newest available assay technology (namely, third-generation TBII assay TRAK Elecsys, the bridge assay IMMULITE, and Mc4-TSI Thyretain bioassay) has comparable cutoff values for the prediction of a severe course of GO. They deliver variable cutoff values during the course of GO for different time points (121). The result of this study is in accordance with a similarly structured study by Jang et al. (2013), who, after comparing the third-generation TBII assay TRAK Elecsys and Mc4-TSI Thyretain bioassay, managed to estimate the risk of severe GO in newly diagnosed GO patients with a disease duration of less than 6 months who did not undergo previous corticosteroid and/or radiotherapy. The researchers determined the cutoff values of the third-generation TBII and Mc4-TSI assays for the prediction of a severe course at 10.67 IU/L and 555.10%, with a specificity of 84.9% and 89.0%, respectively (126). The duration of antithyroid treatment has to be taken into account for applying such cutoff levels, as thyroid therapy influences the TRAb levels significantly. These recent studies have confirmed the results of an earlier publication with a comparable study design where the investigators analyzed the second-generation TBII assay based on the human recombinant TSH-receptor (TRAK human LIA^®^, B.R.A.H.M.S AG, Hennigsdorf/Berlin, Germany) (127, 128).

Lantz et al. (2014) added additional cutoff levels for both TRAB and TSI. Eighty-seven percent of patients who developed GO after the diagnosis of Graves’ hyperthyroidism had TRAb levels above 6.3 IU/L (measured using a human radioreceptor assay kit) (129).

Takakura et al. reported a 14-fold increased risk of developing GO in patients in the top tercile of the initial TSI levels. A TSI index above 400 at the initial presentation may represent a predictor for GO development (130).

The predictive value of the different bioassays is still a main focus in the research field. While some reports have stated that the clinical sensitivity and specificity of TSI for GO are significantly greater than those of TBII (respectively, 97% and 89% versus 77% and 43%) (116), other studies have concluded that both assays have comparable specificities of 89% for TSI and 84.9% for TBII (126). Furthermore, according to the disease stage, the sensitivity of TBII for the prediction of a mild course varied between 51% and 70%, while in the case of a severe course, it reached the maximum of 78% (127).

##### 3.5.1.5 Correlations with clinical activity and bioassays’ predictive power

Multiple studies have observed the positive correlation between TBII and TSI with CAS and severity score (131, 132). Both TBII and TSI correlate with each other and with CAS, but not with GO duration (133, 134). Comparing the two TSI cell-based bioassays [intact wild-type human TSH-R (wt) and the chimeric human TSH-R (Mc4)] with anti-TSH-R binding assays, Mc4/TSI proves to be a better indicator of GO severity and activity. Significant positive correlations are observed between TSI and both CAS and disease severity, while only weak correlations are reported with TRAbs. Furthermore, TSI was positive in all patients with DON and diplopia. All subjects (11 of 200 Graves’ patients) with Mc4-TSI positive/TBII negative assays suffered from severe GO, while seven of 200 Graves’ patients with Mc4-TSI negative/TBII positive titers only had GD without GO, thus suggesting that the sensitivity and specificity may be greater when using TSI bioassays (116, 135). Moreover, TSI was positive in the majority of DON patients, even in clinically inactive subjects, suggesting utility as a marker of disease severity (136). TSI is also measurable in a higher proportion of patients with DON in comparison to the TBII assay (135).

TBII may be an independent risk factor for GO and a potential predictive parameter of disease severity in half of the subjects, as one study showed that there is a 27% higher risk of developing a severe form of disease with each increase of 1 IU/L at 5–8 months after GO onset. Additionally, the prevalence of TBII declined faster in patients with mild GO compared to patients with severe GO; i.e., positivity of TBII after 2 years of disease onset was still observed in 24% of patients with mild course and in 82% of patients with severe disease (127). Higher TRAb serum levels at presentation may be a risk factor for developing GO (129, 135).

The importance of serology in the orbital autoimmune process is also reflected in the positive correlation between the TRAb levels and the severity/activity of GO even after oral corticosteroid therapy (137). Moreover, the higher the TRAb levels, the higher the relapse rate after radiotherapy combined with IVGC (138). Normal TSH and TRAb levels positively affect the therapeutic outcome (73).

#### 3.5.2 Insulin-like growth factor-1 receptor

The IGF1R is directly involved in the pathogenesis of GO (139, 140). IGF1 enhances the actions of the TSHR, and the IGF1R is indirectly activated through cross-talk between IGF1R and TSHR, amplifying the immune response at the level of orbital fibroblasts and increasing GAG production (4). The elucidation of these pathological signaling mechanisms opened the door for direct targeting therapy for GO. Two randomized controlled trials showed that targeting the IGF1R with blocking antibodies led to a significant reduction of proptosis (median 3 mm) and the improvement of eye motility, in addition to inflammation reduction expressed through CAS (91, 141).

It has long been debated whether stimulating IGF1R antibodies contributes to GO, as early *in vitro* experiments indicated such a mechanism (142). However, despite elaborate research efforts, multiple independent groups have failed to detect IGF1R-stimulating antibodies in GO patient sera (4, 143, 144). Other studies that did trace such antibodies in patients’ sera have generally found low prevalence in GO and no correlation with GO features, possibly even proposing a protective effect (145, 146). Therefore, while the IGF1R pathway remains a therapeutically relevant target, the existence of circulating IGF1R autoantibodies as pathogenic or diagnostic biomarkers remains unconfirmed and controversial.

#### 3.5.3 Interleukin-6 and other serological biomarkers

Apart from thyroid-associated biomarkers, other biochemical parameters, often with a relationship to inflammation, have been found to be of potential use in the evaluation of GO and disease activity. Ueland et al. (2022) found higher interleukin-6 (IL6) and colony-stimulating factor 1 (CSF1) levels in serum from GD patients with GO compared to patients without GO (147). Molnar et al. (1997) also described high serum levels of IL6 in GO (148). In addition, raised levels of IL6 have been found in tear fluid from active compared to inactive GO (149). These findings may directly impact treatment decisions, as the inhibition of IL6 function by tocilizumab has proven to be a very effective therapeutic option in patients with steroid-resistant GO (150).

FMS-related tyrosine kinase 3 ligand (FLT3LG) is a cytokine known to be involved in the mobilization and differentiation of hematopoietic stem cells, and it has been linked to autoimmune disorders (151). FLT3LG was higher in patients with severe sight-threatening GO than in moderate GO. In addition, they found elevated fibroblast growth factor 21 (FGF21) levels in patients without signs of GO at baseline but who developed GO later (147).

Among other studied serological biomarkers, soluble IL2 receptor (sIL2R) levels were found to be overexpressed in patients with GO versus control subjects. In addition, higher sIL2R correlates with a higher response rate and a more favorable outcome after corticosteroid therapy compared with patients with normal values (71% versus 47% response rate) (152, 153).

Furthermore, in active patients, high CAS correlates with low selenium levels (154), and concordantly, mean serum selenium decreases when severity increases (155). Based on the theory of orbital tissue remodeling, the serum levels of metalloproteinases and their inhibitors were significantly higher in patients with GO and correlated with CAS, while they decreased after treatment (156). Increased levels of lacrimal oxidative stress biomarkers, 8-hydroxy-2′-deoxyguanosine and malondialdehyde, and of cytokines correlate with CAS and differ significantly in active versus inactive patients (149, 157, 158). Calprotectin, a proinflammatory factor, is overexpressed in both serum and orbital tissue in GO patients and is correlated with CAS (159).

A novelty is the relationship between high serum cholesterol (Cho) and the development of GO, i.e., in patients with a duration of hyperthyroidism less than 4 years, increased levels of total Cho and LDL-Cho correlate with the occurrence of GO (160). Additionally, the correlation between Cho serum levels and CAS raised the possibility of a potential new therapeutic option of statin usage for the treatment of GO (161, 162). A recently published RCT showed that the addition of oral atorvastatin to an IVGC regimen improved GO outcomes in patients with moderate-to-severe, active eye disease who were hypercholesterolemic (163).

Little is known about urinary GAG excretion in GO patients, but some studies have reported a significant elevation in GO patients compared with GD patients without GO (164), in addition to a positive correlation with CAS (165). Despite all these reports about potentially useful parameters, TRAb, regardless of the assay method, remains the best evaluated serological biomarker for activity and severity assessment and prognostic statements.

## 4 Conclusion

Our literature review clearly shows that to this date, there is no other standardized, reliable gold standard for an accurate activity assessment of GO other than CAS. The CAS, despite the fact that it is rather a subjective test, remains the main criterion on which the therapeutic management is based. Even so, we question the validity of CAS due to disputed cases where active orbital inflammation cannot be demonstrated using only clinical signs. In our opinion, imaging techniques, especially MRI, play a critical role in the recognition of early activity signs and in predicting the treatment efficacy of glucocorticoid therapy or radiotherapy through their ability to mirror the edematous changes at the level of orbital soft tissues, thus being able to discriminate subtle subclinical changes that CAS cannot evidence. MRI can provide objective and quantitative parameters of inflammation and is useful in identifying even subclinical and silent orbital changes that could guide the physician in choosing the appropriate treatment for each patient. Nonetheless, it has been shown by recent teprotumumab studies that inflammation and tissue water content are not the only features we should be focusing on when assessing treatment options and contemplating possible responses. Disease severity, including impact on quality of life, but also certain features such as fat-to-muscle ratio on imaging, could guide treatment decisions in the future. MRI also provides detailed structural information and may therefore prove useful to predict treatment outcomes for biologicals. TRAb measurements are as important as imaging, and at the moment, TRAb is the only validated serological biomarker. Sequential measurements inform about autoimmune activity and should be included in the treatment concepts. TSI bioassays are more sensitive and may be more predictive in some patients. Therefore, we emphasize that serological and imaging parameters, i.e., TSI/TBII, respectively, STIR T2 mapping and DWI sequences, reveal decisive information on disease activity even when clinical examination and CAS are not suggestive.

However, an attempt to outline a gold standard is difficult. It should not be defined as one specific assessment but rather as a combination of clinical, serological, and imaging measurements intended to improve the sensitivity of prognosis of disease and response to immunosuppressive, IGF1R, and FcRn blocking or radiation therapy.
